# Lipase Activity in the Larval Midgut of *Rhynchophorus palmarum*: Biochemical Characterization and the Effects of Reducing Agents

**DOI:** 10.3390/insects8030100

**Published:** 2017-09-13

**Authors:** Camilla Camerino Santana, Leandro A. Barbosa, Irinaldo Diniz Basílio Júnior, Ticiano Gomes do Nascimento, Camila Braga Dornelas, Luciano A.M. Grillo

**Affiliations:** 1 Escola de Enfermagem e Farmácia, Universidade Federal de Alagoas, Maceió, AL 57072-900, Brazil; millacamerino@gmail.com (C.C.S.); irinaldodiniz@yahoo.com.br (I.D.B.J.); ticianogn@yahoo.com.br (T.G.d.N.); dornelascb@yahoo.com.br (C.B.D.); 2 Laboratório de Bioquímica Celular, Universidade Federal de São João del Rei, Campus Centro-Oeste Dona Lindu, Divinópolis 35501-296, Brazil; leaugust@yahoo.com.br; 3 Av. Lourival de Mello Mota s/n–CSAU/ESENFAR, Cidade Universitária, Maceió, AL 57072-900, Brazil

**Keywords:** *Rhynchophorus palmarum*, lipase, lipids, midgut, digestion

## Abstract

Lipases have key roles in insect lipid acquisition, storage, and mobilization and are also fundamental to many physiological processes in insects. Lipids are an important component of insect diets, where they are hydrolyzed in the midgut lumen, absorbed, and used for the synthesis of complex lipids. The South American palm weevil *Rhynchophorus palmarum* is one of the most important pests on commercial palm plantations. However, there are few studies about lipid digestion for this insect. In this work, we have described the biochemical characterization of the lipase activity in the posterior midgut of the *R. palmarum* palm weevil. Lipase activity was highest between the temperatures of 37 °C and 45 °C and at pH 6.5. Lipase activity was also sensitive to variations in salt and calcium concentrations. Lipases have been described structurally as enzymes with the Ser-His-Asp Catalytic Triad, containing an active serine. The serine protease inhibitor PMSF (phenylmethane sulfonyl fluoride) inhibited the lipases from *R. palmarum*, demonstrating the importance of a serine residue for this activity. The ability of the lipases to hydrolyze *p*-Nitrophenyl esters with different chain lengths has revealed the activities of a broad range of substrates. The lipase activities of *R. palmarum* increased in the presence of reduced glutathione (GSH) and dithiothreitol (DTT), while in the presence of oxidized glutathione (GSSG), activities were drastically reduced. To our knowledge, this study has provided the first information about lipase activity in the *R. palmarum* palm weevil.

## 1. Introduction

Lipids are essential for all forms of life and they perform an important array of functions in insects. The main lipids in insects are triacylglycerols (TAGs), diacylglycerols (DAGs), phospholipids, hydrocarbons, and wax esters [1]. Triacylglycerols (TAGs) constitute a major lipid component in the diet of insects and their processes of digestion and absorption are very similar to those in mammals [2]. After a meal, TAGs are hydrolyzed in the midgut lumen and the products of digestion are absorbed and used for the synthesis of complex lipids [2,3].

TAG-lipases are enzymes that hydrolyze the outer ester links of TAGs. In insects, these enzymes have more affinity to the unsaturated fatty acids, and they are activated by calcium ions [4,5], thus resembling the actions of mammalian pancreatic lipases. However, there is still quite a difference, as mammals require a co-lipase to bind to lipase in order to fully activate the hydrolysis. Information on the TAG-lipases in insects has been obtained from several species. Smith et al. [6] showed the presence of lipase activities in the gut, where they presumably acted in the utilization of dietary TAGs. Lipases that were purified from the digestive juices of *Bombyx mori*, in addition to their digestive role, showed antiviral activities [7]. TAG-lipase activities have been detected in the midguts of the hemipterans *Rhodnius prolixus* and *Panstrongylus megistus*. Our group showed that at least two TAG-lipase activities were involved in the posterior midgut responsible for dietary TAG hydrolysis and epithelial enzyme-hydrolyzed intracellular TAG pool in the *Rhodnius prolixus* [3]. The role of TAG-lipases in the lipid metabolism of the *P*. *megistus* diet showed that lumenal triacylglycerol was hydrolyzed to mainly fatty acids, glycerol, and, to a lesser extent, DAG [2]. Arrese et al. described the presence and the properties of phosphorylatable TAG-lipases in *Manduca sexta* [8]. In addition, TAG-lipase activities have been observed in the fruit beetle *Pachnoda sinuata* [9].

*Rhynchophorus palmarum* L. (Coleoptera: Curculionidae) feed on the plant species of 12 different families. *R. palmarum* have been reported as one of the most important pests on commercial palm plantations [10] and there are some studies in the literature about their digestive processes and their energetic metabolisms. Bédikou et al. [11] showed α-mannosidase activities from the digestive fluids of *R. palmarum* larvae. Biochemical characterizations for specific β-glucosidases from the digestive fluids of the larvae from *R. palmarum* have been performed [12]. Santana et al. have shown the dynamics of energy source utilization from *R. palmarum* during the embryogenesis [13]. Mobilization kinetics of the lipids and of glycogen and the involvement of TAG-lipases have important roles in the biology of *R. palmarum*. In this paper, we report the presence of lipase activity that may be involved in the digestive processes of the *R. palmarum* insect.

## 2. Materials and Methods

### 2.1. Insects

The larvae (third instar) of *R. palmarum* used in this study were fed exclusively on live vegetative tissue and taken from a colony maintained at 28 °C with 80% relative humidity.

### 2.2. Enzyme Sources

The posterior midguts were dissected from the *R. palmarum* larvae. The luminal content was removed and the tissues were homogenized in a cold buffer (20 mM Tris–HCl, pH 7.4, and containing 0.25 M sucrose) and were used as enzyme sources. The protein concentrations were determined as described by Bradford using bovine serum albumin as a standard [14].

### 2.3. Lipase Activity

The lipase activities were assayed as described by Choi et al. [15]. The standard reaction mixture (10 mM 2,3-dimercapto-1-propanol tributyrate (DMPTB), 40 mM 5,5′-dithiobis(2-nitrobenzoic acid) (DTNB), 0.5 M ethylenediaminetetraacetic acid, 10% Triton X-100, and 1 M Tris-Cl, pH 7.5) was prepared in a microcentrifuge tube. Microplate wells were filled with 180 µL of this mixture, and 20 µL (10 µg) of the enzyme samples were added to each well. The reaction was incubated at 37 °C for 30 min and the absorbance was measured at 405 nm using a Flex Station III microplate reader (Molecular Devices, Sunnyvale, CA, USA). We used a blank that contained no DMPTB. In the DMPTB–DTNB method, free thiol groups that are generated by the lipase hydrolysis of DMPTB reduce DTNB to create a yellow color. Three samples were analyzed for each experimental point. The assay was read to an end point and the molar extinction coefficient of DTNB 13.6 M^−1^ cm^−1^ was used for calculations.

### 2.4. Effects of pH, Calcium, and Salt Concentrations

The lipase activity was measured at different pH values utilizing a Tris-HCl buffer (1 M—pH 7.0–9.0) and a sodium acetate buffer (1 M—pH 5.5–6.5). The effect of salt concentration on lipase activity was evaluated through the incubation of homogenates in buffers containing different concentrations of NaCl (0–1.5 M). The calcium requirements were tested by using a reaction buffer containing 1 mM EGTA, and CaCl_2_ was added in order to obtain the desired range of [Ca^2+^]-free concentrations (0.5–1.5 M).

### 2.5. PMSF Effect

Increasing concentrations of phenylmethane sulfonyl fluoride (PMSF) (Sigma-Aldrich Co, St Louis, MO, USA) were added to the reaction mixtures (described in Section 2.3) and the lipase activity was determined.

### 2.6. Substrate Specificities

The lipase hydrolytic specificities were assayed with the chromogenic *p*-Nitrophenyl ester substrates: *p*-Nitrophenyl caprylate (C8), *p*-Nitrophenyl decanoate (C10), *p*-Nitrophenyl laurate (C12), and *p*-Nitrophenyl palmitate (C16) (Sigma-Aldrich Co, St Louis, MO, USA). The reaction mixtures contained 1 mL of the cold buffer (20 mM Tris–HCl, pH 7.4, 0.25 M sucrose) and 100 μL substrate (3 mM *p*-Nitrophenyl ester in 2-propanol). The reactions were started by the addition of the substrate at 37 °C, followed by an incubation at 37 °C for 30 min. The *p*-Nitrophenyl that was released was monitored at 410 nm. A standard curve of *p*-Nitrophenyl-esters was used to calculate the activity.

### 2.7. Effects of the Reducing Agents

To determine the effects of reducing agents on lipase activity, reduced glutathione (GSH), dithiothreitol (DTT), and oxidized glutathione (GSSG) (Sigma-Aldrich Co, St Louis, MO, USA) were incubated with the posterior midgut tissue homogenates for 30 min with different concentrations of each reagent. The lipase activities were measured with *p*-Nitrophenyl palmitate.

### 2.8. Statistical Analyses

The data are expressed as the mean ± standard deviation (SD). The statistical comparisons were made by one-way ANOVA with a post-test. The significant level was set at * *p* < 0.05.

## 3. Results

**Figure 1:**
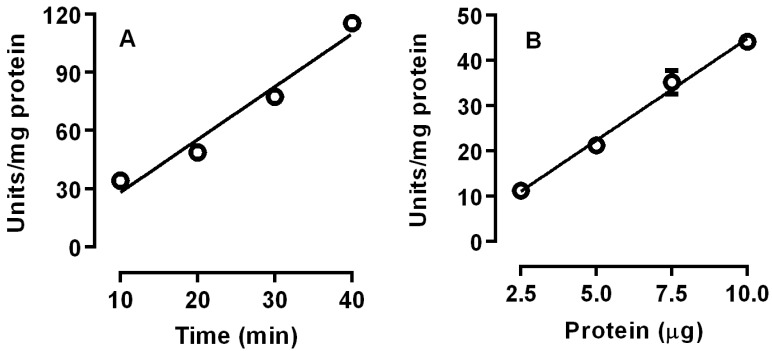
Time course (**A**) and the effects of the enzyme concentrations (**B**) on the lipase activity of the posterior midgut homogenate (crude extract) of *R. palmarum* larvae. The error bars represent the standard deviation (SD) for three determinations.

**Figure 2:**
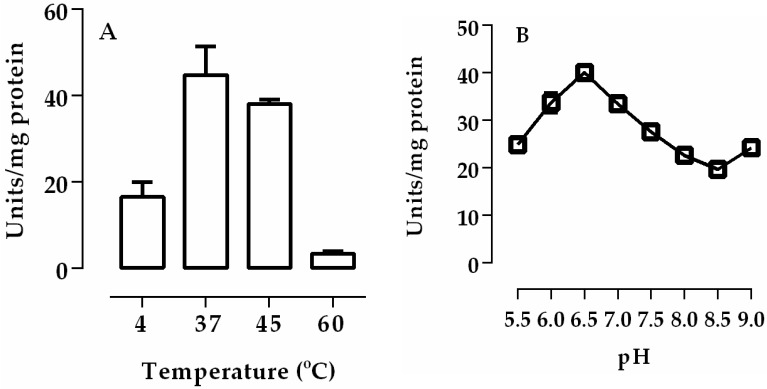
The optimum temperature (**A**) and optimum pH (**B**) for lipase activity of the posterior midgut homogenate (crude extract) of *R. palmarum* larvae. The error bars represent the SD for the three determinations.

We investigated the time-course and the effects of protein concentrations of lipase activity of posterior midgut homogenate (crude extract) of *R. palmarum* larvae. Linear values were obtained for lipase activities during the entire 40 min incubation, indicating that they were stable under the conditions that were used. In addition, when the protein concentrations were increased, a proportional increase in linear activities was observed, indicating that the activity of the enzyme source and the incubation conditions were adequate (Figure 1). The temperatures and the pH of the incubation conditions were critical to lipase activity. Lipases in the posterior midguts displayed a maximum activity at 37 °C and at pH 6.5 (Figure 2).

**Figure 3:**
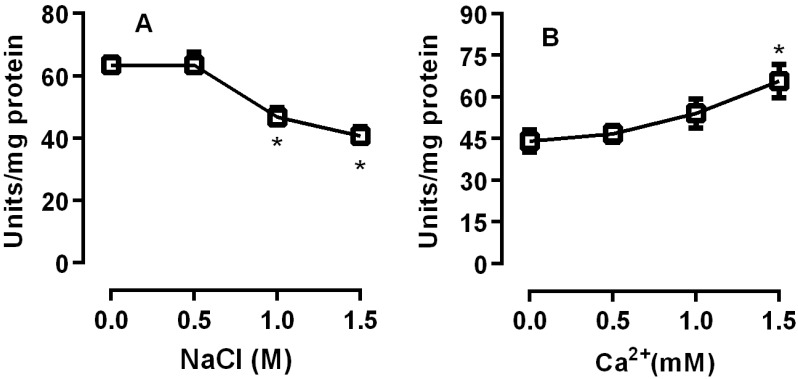
Effects of NaCl and calcium concentrations on the lipase activities of the posterior midgut homogenate (crude extract) of *R. palmarum* larvae. The incubations were performed with different NaCl concentrations (**A**) and with different calcium concentrations (**B**). The error bars represent the SD for the three determinations (* *p* < 0.05).

**Figure 4:**
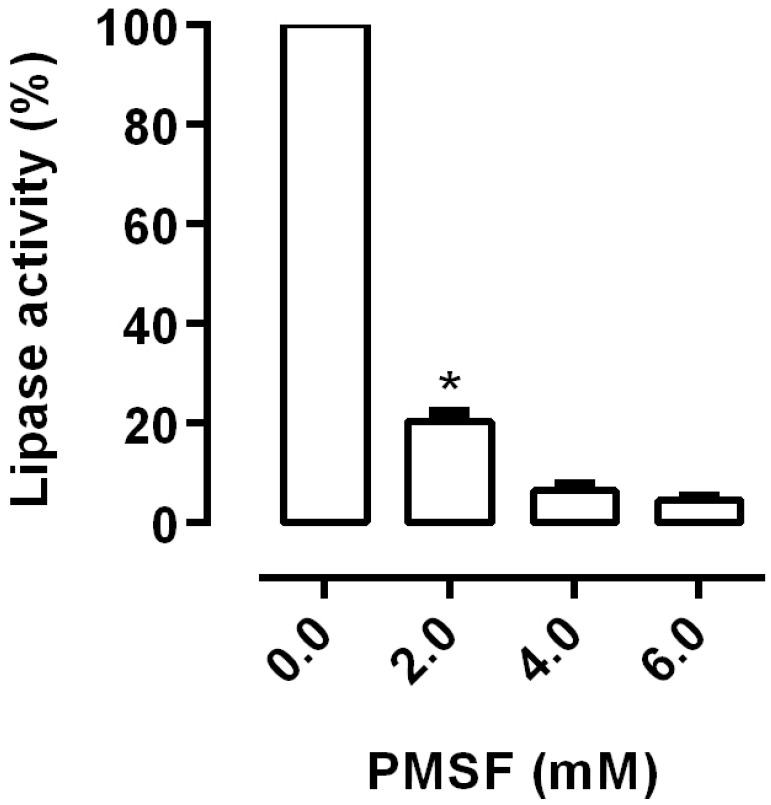
Effects of phenylmethane sulfonyl fluoride (PMSF) on the lipase activities of the posterior midgut homogenate (crude extract) of *R. palmarum* larvae. The incubations were performed with different PMSF concentrations. The error bars represent the SD for the three determinations (* *p* < 0.05).

The effect of ionic strength on lipase activity was assessed. Salt concentrations higher than 0.5 M reduced lipase activity (*p* < 0.05). In contrast, the presence of calcium increased the lipase activity, with a significant increase at 1.0 and 1.5 M NaCl (*p* < 0.05) (Figure 3). Structurally, lipases contain a Ser-His-Asp Catalytic Triad in which the serine residue is catalytically important. This active serine is part of the sequence GXSXG commonly found in lipase sequences [16,17]. PMSF is a synthetic serine protease inhibitor that has been used to evaluate the role of the serine residues on lipase activity. The necessity of this active serine for lipase activity in *R. palmarum* was assessed through the incubation of PMSF with the homogenates. PMSF inhibited 80% of the lipase activity from *R. palmarum*, demonstrating the importance of a serine residue for this activity (Figure 4).

**Figure 5:**
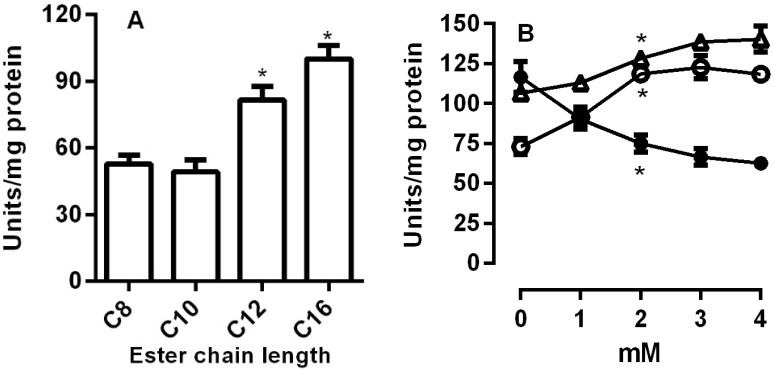
Substrate specificity and the effects of the reducing agents on the lipase activity. The posterior midgut homogenate (crude extract) of *R. palmarum* was incubated with the *p*-Nitrophenyl esters with different chain lengths (**A**). The effects of reducing agents (**B**) were determined by incubation with different concentrations of reduced glutathione (GSH) (open circles), dithiothreitol (DTT) (open triangles), and oxidized glutathione (GSSG) (filled circles). Lipase activity was measured using C16 pNP-palmitate as substrate. The error bars represent the SD for the three determinations (* *p* < 0.05).

Next, we observed the abilities of the lipases from *R. palmarum* to hydrolyze different chain lengths of *p*-Nitrophenyl esters. The results demonstrated that the lipase activity increased with the longer fatty acid chains (Figure 5A).

The thiol groups are important for the lipase activities in the fat bodies of *Manduca sexta* [18]. In order to investigate the importance of thiol groups in *R. palmarum*, homogenates were incubated in different concentrations of GSH, GSSG, and DTT. Lipase activities were increased in the presence of thiol groups and were drastically reduced in the presence of GSSG (open squares (Figure 5B).

## 4. Discussion

Digestion of ingested food is a process by which nutrients are acquired by insects, and if we understand the physiology of insect digestion, it will be possible to establish possible targets for insect control [18]. *R. palmarum* is one of the most important pests of commercial palm plantations [10]. However, there are no studies concerning the digestion of lipids for this insect. The present work evaluated the biochemical characterization of lipase activity in the posterior midgut of *R. palmarum* larvae. The activity was linear, indicating that the enzyme source and the incubation conditions were adequate (Figure 1).

Lipases in the posterior midgut of *R. palmarum* larvae showed the highest activity at pH 6.5 (Figure 2). Most insects have the highest enzyme activity registered between pH 5.5 and 7.5. The lipases of *L. migratoria* showed an optimum activity at pH 6.9 and *R. prolixus* showed two peaks of activity with optimum pH being between 5.5 and 7.5 [3,19]. Zibaee et al. showed the optimal pH for lipase activity in the midgut of *C. suppressalis* and *N. aenescens* [20,21]. The soluble and membrane-bound digestive lipases of the midgut of *P. brassicae* showed an optimal pH of 8 and 10, respectively [22]. For *R. palmarum* palm weevils, digestive juices showed an optimum pH for β-glucosidase and α-mannosidase activity between 4.5 and 6.0 [11,12]. We observed a decrease of 20–25% in activity at salt concentrations higher than 0.5 M (Figure 3A). Similar results were observed for lipases from *Cochliomyia hominivorax* [23,24]. In contrast, calcium increased lipase activity by 15–20% at concentrations of 1.0 and 1.5 M (Figure 3B). This dependence was also shown for enzymes from *L. migratoria* and *R. prolixus* [3,23].

Lipases typically contain the Ser-His-Asp Catalytic Triad. The triad is similar to other catalytic sites of several enzymes in the α/β hydrolase-fold superfamily and enhances the nucleophilic potential of serine oxygen, which is the key residue involved in the reaction with substrate [16,17]. The serine blocker PMSF inhibited the lipase activity from *R. palmarum*, suggesting the importance of a serine residue for this activity (Figure 4). A similar result has also been shown for the lipases from *Phlebotomus papatasi* and *R. prolixus* [3,25]. We also assessed the optimum chain length of fatty acid esters for *R. palmarum* lipase activity. While lipases isolated from the labial glands of *Bombus terrestris* showed a broad range of activities to the fatty acid esters with medium chain lengths [23], our results have shown the highest activity with medium and long chained fatty acid esters (Figure 5A).

Lipases from the α/β hydrolase family have a conserved Cys 130 and Cys 234 that flank the lid region of the enzyme. The lid, which covers the active site, rotates to allow the interaction with the substrate. These Cys residues are sensitive to thiols and are involved in the mechanism of lipase activation [18]. The presence of thiol groups, via increasing concentrations of GSH, GSSG, and DTT, was observed to affect lipase activities, indicating that lipase activities are sensitive to redox status (Figure 5B). The thiol groups that enhanced lipase activities were in the reduced-state. The triglyceride lipases from the fat bodies of *M. sexta* showed the same effects, and the authors explained that GSSG would form a mixed disulfide with the lipase thiol groups. This explained the decreased of its activity, similar to that found with other proteins [18].

The present study identifies, for the first time, a lipase activity in the posterior midgut of *R. palmarum* that may be involved in the lipid digestion of this insect. Lipase showed that the highest activity at pH 6.5 was sensitive to salt concentrations. The serine protease inhibitor PMSF inhibited the activity, demonstrating the importance of a serine residue at the active site. This enzyme has a broad range of activities to fatty acid esters with medium and long chain lengths. Furthermore, the thiol groups in reduced-state were important for the activity.
